# High Pectin Recovery from Cocoa Husks Using an Autoclave Approach: An Analysis of Its Physicochemical, Structural, and Genotoxicity Properties

**DOI:** 10.3390/foods13050669

**Published:** 2024-02-22

**Authors:** Thanaporn Pinkaew, Woorawee Inthachat, Chanakan Khemthong, Varongsiri Kemsawasd, Nattira On-Nom, Piya Temviriyanukul

**Affiliations:** 1 Master of Science Program in Toxicology and Nutrition for Food Safety, Institute of Nutrition, Mahidol University, Nakhon Pathom 73170, Thailand; thanaporn.pik@student.mahidol.ac.th; 2 Food and Nutrition Academic and Research Cluster, Institute of Nutrition, Mahidol University, Salaya, Phuttamonthon, Nakhon Pathom 73170, Thailand; woorawee.int@mahidol.ac.th (W.I.); chanakan.khe@mahidol.ac.th (C.K.); varongsiri.kem@mahidol.ac.th (V.K.); piya.tem@mahidol.ac.th (P.T.)

**Keywords:** agricultural waste, cocoa, cocoa husk, genotoxicity, pectin, physicochemical properties, response surface methodology, sustainability

## Abstract

Pectin was extracted from cocoa husks, a food-processing biowaste, using an autoclave approach. A Box–Behnken design (BBD) and response surface methodology (RSM) were used to optimize pectin extraction. Three factors including extraction time (5–40 min), temperature (105–135 °C), and solid to liquid ratios (SLRs) (10–30 *w*/*v*) were employed. Results showed that the optimal conditions for high cocoa-husk-pectin (CHP) yield of 26.22% was 105 °C for 5 min with an SLR at 20 *w*/*v*. The physicochemical characteristics of CHP were compared with commercial high-methoxyl pectin (CHMP) and commercial low-methoxyl pectin (CLMP). CHP was classified as low-methoxyl pectin, with a degree of esterification at 34.74% and methoxyl content of 5.08%. The galacturonic acid content of CHP was 32.71% which was lower than CHMP (72.69%) and CLMP (41.24%). The intrinsic viscosity and viscosity–average molecular weight was similar to CLMP but higher than CHMP. No significant differences in water-holding capacity were found among samples. CHP showed higher oil-holding capacity but lower solubility compared with commercial pectin. CHP solutions showed pseudoplastic behavior. The viscosities of CHP solutions improved at increasing concentrations and decreasing pH. The CHP solution viscosities were lower than CLMP at the same condition. The viscoelastic properties of CHP solutions increased at higher concentrations, with the optimal value at pH 3. CHP showed no genotoxicity when assayed using the Ames test. Autoclave extraction as an accessible fast method showed potential for high pectin yield recovery from cocoa husks.

## 1. Introduction

*Theobroma cacao* L. (family Sterculiaceae), or the cacao tree, is one of the most important global crops. Cocoa beans are used to produce chocolate, snacks, and beverages. The cocoa pod husks or cocoa bean shells, comprising approximately 75% of the cocoa fruit, are removed during processing [1]. This waste by-product is often discarded in the cocoa plantation, producing foul odors, and is a possible source of the botanical disease inoculum causing black pod rot [2,3], while the high levels of organic acids in cocoa husks increase soil acidity and contaminate the environment [4]. The cocoa market is forecasted to expand at a compound annual growth rate (CAGR) of 7.33% up to 2025, resulting in increased quantities of cocoa husks. Cocoa husks contain many active chemicals, including phenolics, flavonoids, alkaloids, theobromine, methylxanthines, dietary fibers, and pectin. These compounds are all potential ingredients in the nutraceutical, cosmetics, and food industries. Therefore, to add economic value to cocoa husks and support the bio-circular economy, this study investigated the recovery of pectin from this biowaste using an autoclave-based extraction method.

Pectin is a natural polymer consisting of a chain of galacturonic acid units linked with α-(1→4) glycosidic bonds. Pectin is used for many food applications including as a gelling agent, fat replacer, emulsifier, thickener, and stabilizer [5]. Pectin also has been used in the pharmaceutical industry and in other applications such as for developing edible films, paper substitutes, foams, and plasticizers. This was due to its easy accessibility, non-toxic properties, and cheap cost of production [6]. It has been reported that there is to be expected a 5–6% increase in the market demand for pectin [7]. Major sources of pectin are industrial by-products, such as citrus peels and apple pomace [8], with mango peel [9], durian rind [10], and cocoa husks [11] also being used for pectin extraction. Strong acids such as ascorbic acid, citric acid, hydrochloric acid, nitric acid, and oxalic acid are typically employed in the pectin-extraction process, resulting in high pectin recovery and galacturonic acid content [12]. However, when poorly managed, strong organic acids can cause a significant adverse impact on the environment and long extraction times are required.

Several extraction methods have been used to recover pectin from plant sources including enzymatic extraction, and ultrasound-, microwave-, thermal- and autoclave-assisted extraction [13]. Pectin extraction from cocoa husks using hot water extraction at 50 °C for 90 min and at 100 °C for 90 min gave 7.5% and 12.6% pectin yield, respectively [14], implying that high temperatures enhanced pectin recovery. Microwave-assisted pectin extraction using citric acid as the solvent at 300 Watt (W) for 30 min gave pectin recovery of 3.51%, while six-fold-increased pectin yields were obtained after extraction with citric acid solvent at 450 W and 104 °C for 30 min [15,16]. These results suggested that the autoclave (high temperature) approach may be suitable for pectin extraction from cocoa husks, presumably by promoting plant cell rupture. To the best of our knowledge, no application currently exists for the extraction of cocoa husk pectin (CHP) using an autoclave approach. Although, autoclave-assisted extraction may not be entirely deemed an eco-friendly approach, since this method consume high amounts of energy to generate pressure and heat. In contrast, autoclave extraction is a rapid procedure that is easily accessible in both laboratory and industrial settings. Furthermore, pectin yields are not significantly impacted by various extraction conditions when an autoclave is used compared to other methods, since only some factors can be adjusted, including temperature, time, and solid to liquid ratios (SLR). The autoclave method makes regulating pectin production simple and no potent organic acids have to be utilized.

This study extracted CHP using an autoclave method using a Box–Behnken design (BBD) and response surface methodology (RSM) to optimize the extraction conditions. Three extraction factors including extraction time, extraction temperature, and solid to liquid ratios were employed. Pectin extracted under the optimal condition was further investigated for physicochemical characteristics and compared with commercial pectin. Genetic toxicity was also analyzed using the bacterial reverse mutation test (Ames test). The results provide insights into the physicochemical characteristics of CHP using an autoclave extraction method.

## 2. Materials and Methods

### 2.1. Sample Preparation and Chemicals

Fresh cocoa husks (*Theobroma cacao* L.) utilized in this work were obtained from Tanatip Farm in Prachuap Khiri Khan, Thailand. The cocoa husks were cut into small pieces and washed with distilled water. The washed cocoa husks were dried using a Binder GMBH hot air oven (model FFD115/E2, Binder GmbH, Tuttlingen, Germany) at 60 °C for 48 h; they were ground in the blender (Phillips 600W series, Philips Electronics Co., Ltd., Jakarta, Indonesia), and passed through a 60-mesh sieve, respectively. The obtained powder was packed in an aluminum foil bag and kept at −20 °C before the experiment analysis. The moisture content of the cocoa husk powder was determined using a Halogen HE53 moisture analyzer (Mettler-Toledo AG, Greifensee, Switzerland) and represented as 3.45 ± 0.26%. The color was performed in CIELAB units as 49.01 ± 0.04 for L*, 11.05 ± 0.01 for a*, 24.16 ± 0.00 for b* using a ColorFlex EZ Spectrophotometer (Hunter Associates Laboratory, Reston, VA, USA). Moreover, pesticide residues and heavy metals in cocoa husk powder were analyzed at Bureau Veritas AQ Lab (Pathumthani, Thailand) according to the in-house method TPTFS-229TM and TPT-FS-282TM based on AOAC [17], respectively.

The commercial high-methoxyl pectin (CHMP) extracted from red apples was purchased from Chemipan Corporation Co., Ltd., Bangkok, Thailand. Moreover, the commercial low-methoxyl pectin (CLMP) extracted from fruit pulp was obtained from Louis François Sas, Marne La Vallee Cedex 2, France. All chemicals and reagents utilized in this investigation were obtained from Sigma-Aldrich (St. Louis, MO, USA) and were of analytical grade.

### 2.2. Pectin Extraction

Cocoa husk pectin was autoclave extracted as previously described with some modifications [18]. After pectin extraction, the slurry was strained through two layers of muslin cloth, and the filtrate was mixed with 95% ethanol (1:1, *v/v*) to precipitate the pectin. The precipitated pectin was left for 15 h at room temperature (25 ± 2 °C) and then isolated using centrifugation at 4600× *g* for 20 min before washing with 50% acetone and drying at 60 °C for 4–8 h in a hot-air oven. The dried pectin was ground using a blender and passed through a ≥120-mesh sieve, with the pectin yield calculated as follows:

$$\mathrm{Pectin}\;\mathrm{yield}\;(\%)=\frac{\mathrm{Dried}\;\mathrm{pectin}\;(g)}{\mathrm{Initial}\;\mathrm{dry}\;\mathrm{powder}\;\mathrm{of}\;\mathrm{cocoa}\;\mathrm{husk}\;(g)}\times 100$$

### 2.3. Experimental Design

A Box–Behnken design (BBD) with RSM was utilized in this work to optimize and examine the impact of process variables (time (X_1_), temperature (X_2_) and solid to liquid ratio (SLR) (X_3_)) on the responses (extraction yield). These factors and their levels are shown in Table 1. The relationship between process variables and responses was expressed using the empirical second order polynomial equation as follows:

$$\mathrm{Yield}\;(\%)=a_{0}+\sum_{i=1}^{3}a_{i}X_{i}+\sum_{i=1}^{3}a_{\mathrm{ii}}X_{i}^{2}+\sum_{i=1}^{2}\sum_{j=2}^{3}a_{\mathrm{ij}}X_{i}X_{j}$$

where a_0_ is a constant coefficient; a_i_, a_ii_ and a_ij_ are the coefficients of the linear, quadratic and interaction terms, respectively.

### 2.4. Physicochemical and Structural Characteristics of Pectin

#### 2.4.1. Color, pH, Moisture Content, and Water Activity (a_w_)

Color was analyzed using a Colorflex EZ Spectrophotometer (HunterLab, Reston, VA, USA) and reported in a CIELAB unit as L* (lightness), a* (redness), and b* (yellowness). The pH measurement was performed using a pH meter (model FE20-Kit FiveEasy^TM^, Mettler Toledo, Temecula, CA, USA). The moisture content was determined using a moisture balance instrument (model MOC63U, Shimadzu, Nakagyo, Kyoto, Japan), and water activity (a_w_) was measured using a water activity meter (model ms1, Novasina, Lachen, Switzerland).

#### 2.4.2. Equivalent Weight and Methoxyl Content (MeO,%)

Equivalent weight was determined as previously described [19]. In brief, 0.5 g of the dried pectin sample was placed in a 250 mL conical flask, and 10 mL of deionized water was added. One gram of sodium chloride was added to this mixture to sharpen the titration endpoint, followed by three drops of phenol red indicator using 0.1 N NaOH. The solution was titrated until a pink color appeared. The equivalent weight was calculated using the following formula:

$$\mathrm{Equivalent}\;\mathrm{weight}\;(\mathrm{mg})=\frac{\;\mathrm{Pectin}\;\mathrm{sample}\;(g)\times 1000}{\mathrm{Volume}\;\mathrm{of}\;\mathrm{NaoH}\;\left(\mathrm{mL}\right)\times \mathrm{Normality}\;\mathrm{of}\;\mathrm{NaoH}\;(N)}$$

Methoxyl content (MeO,%) was determined based on the equivalent weight of the neutral solution following the method of Chaliha et al. [20]. Then, 25 mL of 0.25 N NaOH was added to the neutralized solution, stirred, and left at room temperature for 30 min. Subsequently, 25 mL of 0.25 N HCl was added to neutralize NaOH, and the sample was shaken until the pink color turned yellow. Titration against 0.1 N NaOH was then conducted using three drops of phenol red indicator to determine the titration endpoint. The methoxyl content was calculated as follows:

$$\mathrm{Methoxyl}\;\mathrm{content}\;(\%)=\frac{\mathrm{Volume}\;\mathrm{of}\;\mathrm{NaOH}\;\left(\mathrm{mL}\right)\times \mathrm{Normalityof}\;\mathrm{NaOH}\;(N)\times \;3.1}{\mathrm{Pectin}\;\mathrm{sample}\;(g)}$$

#### 2.4.3. Degree of Esterification (DE,%) and Anhydrouronic Acid Content (AUA,%)

Following Shivamathi et al. [21] and Liu et al. [22], 0.5 g of dried pectin sample was dissolved in 2 mL ethanol and 100 mL deionized water. The mixture was continuously stirred until complete solubility was achieved. Five drops of phenolphthalein indicator were added to the solution, and 0.5 N NaOH was titrated until a pink color appeared (V_1_). Subsequently, 10 mL of 0.5 N NaOH was added to the titrated sample, stirred for one minute, and then left to stand at room temperature for 15 min. Following this, 10 mL was mixed with 0.5 N HCl and stirred until a colorless solution formed. A phenolphthalein indicator was added and the solution was titrated again using 0.5 N NaOH until a pink color appeared (V_2_). The degree of esterification content (%DE) was calculated using the formula:

$$\mathrm{DE}\;(\%)=\frac{V_{2}\;(\mathrm{mL})}{V_{2}\left(\mathrm{mL}\right)+V_{1}\;(\mathrm{mL})}\times 100$$

The DE also was analyzed using Fourier-transform infrared (FTIR) spectroscopy, as described by Maneerat et al. [23]. Dried pectin samples were positioned on the attenuated total reflectance sampling accessory (Smart iTR, Thermo Fisher Scientific, Waltham, MA, USA) of an FTIR spectrometer (Nicolet 6700, Thermo Fisher Scientific). FTIR spectra were recorded at a resolution of 4 cm^−1^ with 64 scans ranging from 400 to 4000 cm^−1^. The DE was calculated using the following formula:

$$\mathrm{DE}\;\left(\%\right)=87.609\;\times \left(\frac{{\mathrm{Area}}_{1}}{{\mathrm{Area}}_{1}+{\mathrm{Area}}_{2}}\right)+25.768$$

In this context, Area_1_ and Area_2_ denote the regions under the peaks observed at wavelengths of 1760–1745 and 1640–1620 cm^−1^, respectively.

$$\mathrm{Anhydrouronic}\;\mathrm{acid}\;\left(\%\right)=\frac{176\;\times \;0.1y\;\times \;100}{W\;\times \;1000}+\frac{176\;\times \;0.1z\;\times \;100}{W\;\times \;1000}$$

Anhydrouronic acid (AUA) content was determined as previously reported [19] and calculated using the following formula.

Where y is the volume of NaOH from the equivalent weight determination (mL), z is the volume of NaOH from methoxyl content determination (mL), and w is the weight of the pectin sample (g).

#### 2.4.4. Intrinsic Viscosity and Viscosity–Average Molecular Weight (M_v_)

The intrinsic viscosity [ƞ] of pectin was determined as described by Maneerat et al. [23]. Pectin solutions at concentration ranges of 0.025% to 0.2% (*w*/*v*) were prepared and measured for intrinsic viscosity using a Cannon-Fenske Viscometer (Capillary No. 50, internal diameter 0.44 mm; Schott-Geräte, Hofheim, Germany) at 30 °C. The intrinsic viscosity was estimated by extrapolating the value of the specific viscosity from Huggins and Kraemer plots to zero concentrations from 0.025% to 0.2% (*w*/*v*). The viscosity–average molecular weight (M_v_) was calculated from the Mark–Houwink–Sakurada equation:

$$[\eta ]=K{\left(M_{v}\right)}^{\alpha }$$

where [ƞ] is the intrinsic viscosity (cm^3^/g), and K and α are temperature-depending parameters, constants of solute, and solvent characteristics, which are K = 9.55 × 10^−4^ (g/dL) and α = 0.73, respectively [24].

#### 2.4.5. Water- and Oil-Holding Capacity (WHC and OHC)

Water- and oil-holding capacity were determined following the method of Kazemi et al. [25]. In brief, 1 g of dried pectin sample was mixed into 10 mL of distilled water, then vortexed for 1 min and centrifuged at 3000× *g* for 30 min. The supernatant was discarded, and the residue was weighed. The oil-holding capacity was measured using sunflower oil with a 0.9 g/mL density instead of distilled water with the same measurement process. The WHC and OHC were calculated using the following formula:

$$\mathrm{Water}-\mathrm{holding}\;\mathrm{capacity}=\frac{\mathrm{Weight}\;\mathrm{of}\;\mathrm{precipitate}\;\mathrm{sample}\;(g)}{\mathrm{Pectin}\;\mathrm{sample}\;(g)}$$

$$\mathrm{Oil}-\mathrm{holding}\;\mathrm{capacity}=\frac{\mathrm{Weight}\;\mathrm{of}\;\mathrm{precipitate}\;\mathrm{sample}\;(g)}{\mathrm{Pectin}\;\mathrm{sample}\;(g)}$$

#### 2.4.6. Water Solubility Index (WSI)

The water solubility index at different pH levels was determined following Begum et al. [26]. In summary, 3 g of dried pectin sample was dispersed in 100 mL of deionized water and adjusted to pH 2, 4, 6, 8, or 10 using either 0.1 M HCl or 0.1 M NaOH. The supernatant of the pectin was used to determine pectin solubility after centrifugation at 2700× *g* for 15 min.

$$\mathrm{Solubility}\;(\%)=\frac{\mathrm{Weight}\;\mathrm{of}\;\mathrm{dried}\;\mathrm{supernatant}}{\mathrm{Weight}\;\mathrm{of}\;\mathrm{dried}\;\mathrm{sample}}\times 100$$

#### 2.4.7. Rheological Measurements

The rheological properties of the dried pectin samples were assessed for their flow behavior and the viscoelasticity of pectin solutions at various concentrations and pH levels. Pectin solutions were prepared in distilled water at different concentrations (1.0%, 1.5%, 2.0%, 2.5%, and 3.0% *w*/*v*). Pectin solutions at 3.0% *w*/*v* were also prepared at different pH values (2, 3, 4, 5, and 6) using hydrochloric acid or sodium hydroxide solutions. All pectin solutions were refrigerated at 4 °C overnight before analyses [10]. The rheological properties were measured at 25 °C using a rheometer (HAAKE^TM^ MARS^TM^, 40 Rheometer, Thermo Fisher Scientific Inc., Waltham, MA, USA,) equipped with a cone and plate sensor (cone diameter: 35 mm, angle: 2°, gap: 0.100 mm). The flow behavior analysis was performed by programming the cone of the measuring sensor to linearly increase the shear rate from 1 s^−1^ to 100 s^−1^.

Strain sweep (0.01–100%) testing at a fixed frequency of 10 rad/s was used to determine the linear viscoelastic range (LVR). Dynamic frequency sweep tests (from 0.1 to 100 rad/s) were performed with a constant strain of 0.5% within the linear region. The mechanical characteristics of the emulsions were measured by recording the storage modulus (G′) and the loss modulus (G″) as functions of frequency.

### 2.5. Genotoxicity Analysis of CHP

The genotoxicity test was conducted following the OECD guidelines ‘Bacterial Reverse Mutation Test’ No. 471 (Ames test) [27]. CHP (10–2000 µg/plate) was incubated with *Salmonella typhimurium*, including TA 98, TA100, TA102, TA1535, and TA1537, in the presence or absence of S9 extract [28]. The S9 extract (Sigma-Aldrich, St. Louis, MO, USA) was used to detect indirect-acting mutagens. Positive controls 4-nitroquinoline-1-oxide (4NQO, 0.2 µg/plate for TA98), sodium azide (NaN_3_, 2.5 µg/plate for TA100 and 0.5 µg/plate for TA1535), mitomycin C (MMC, 0.5 µg/plate for TA102), and 9-aminoacridine (9-AA, 50 µg/plate for TA1537) were used in the experiment without S9 extract, while, 2-aminoanthracene (2-AA, 2 µg/plate) was used in the experiment with S9 extract. The mutagenicity ratio (MR) was calculated as previously explained [29,30].

### 2.6. Statistical Analysis

All experiments were determined in triplicate, and the data were shown as mean ± SD (*n* = 3). A one-way analysis of variance (ANOVA) was analyzed using Duncan’s multiple range test using SPSS version 18 (Statistical Package for the Social Sciences, SPSS Inc., Chicago, IL, USA). The data were considered to be significantly different at *p* < 0.05. The BBD and RSM were assigned using Design-Expert version 13 (Stat-Ease Inc., Minneapolis, MN, USA).

## 3. Results and Discussions

### 3.1. Accuracy and Variance of the Regression Model

A Box–Behnken design (BBD) was performed to investigate the effect of the three independent variables (time (X_1_), temperature (X_2_), and solid to liquid ratios (SLR) (X_3_)) on responses (extraction yield of CHP). Using the BBD (Table 1, Table 2 and Table 3), 15 runs for nine factorial points, three axial points, and three center points were carried out, with CHP extraction yields ranging from 1.85 to 26.15% (Table 3).

The analysis of variance (ANOVA) results of the regression model are shown in Table 4. The ANOVA results showed whether the model variations were significant compared to the variability of the results obtained in the laboratory. The coefficient of determination (R^2^) and adjusted determination coefficient (adjusted R^2^) were used to determine the adequacy of the obtained model. The R^2^ of CHP yield was 0.9781, while the adj R^2^ also was high (0.9386). The *p*-value of the model was significant (0.0013), while the lack of fit was insignificant (0.0547). According to the results, the acquired model can account for the majority of the variance in the CHP extraction yield [31,32]. The relationship between the response (CHP yield) and independent variables (time (X_1_), temperature (X_2_), and SLR (X_3_)) are shown below:

Yield (%) = 173.34 − (0.426X_1_) − (1.895X_2_) − (0.401X_3_) − (0.001X_1_X_2_) − (0.006X_1_X_3_) − (0.002X_2_X_3_) + (0.012X_1_^2^) + (0.006X_2_^2^) + (0.011X_3_^2^)

### 3.2. Effect of Independent Variables on Cocoa Husk Pectin (CHP) Yield

**Figure 1:**
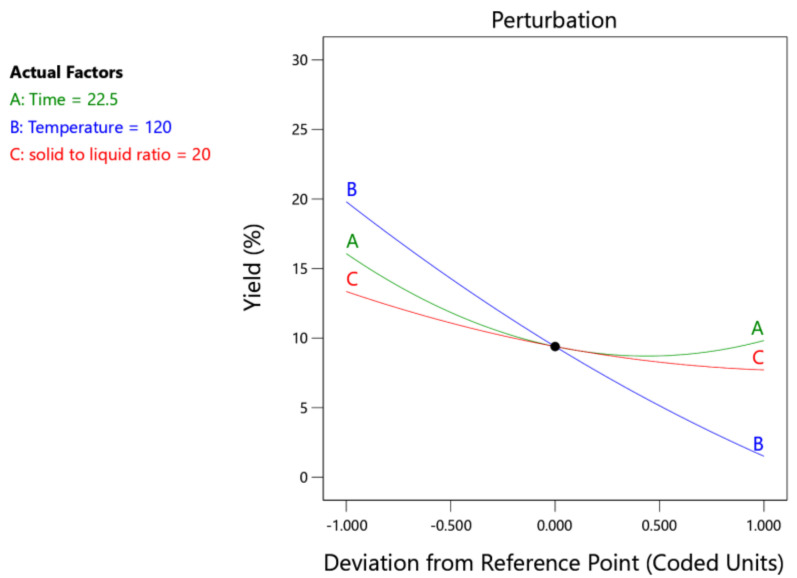
Perturbation plot for extraction yield of cocoa husk pectin (A−time; B−temperature; C−SLR).

Figure 1 shows a perturbation plot for the extraction yield of CHP to assist in finding the variables that had the greatest effect on the response. The perturbation graph showed that the extraction temperature played the greatest role in the production of pectin from cocoa husks. Maran et al. [33] stated that a steep slope for a factor indicated that the response was sensitive.

**Figure 2:**
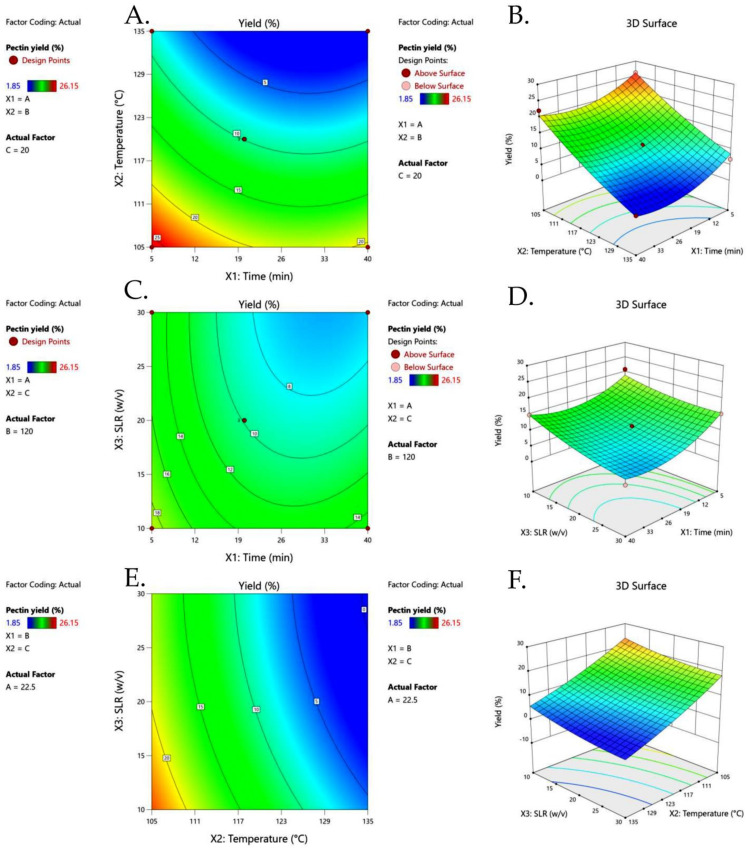
Contour plots (**A**,**C**,**E**) and response surface plots (**B**,**D**,**F**) of extraction yield of cocoa husk pectin affected by extraction time (X_1_), extraction temperature (X_2_), and solid−liquid ratio (SLR) (X_3_).

The contour and three-dimensional (3D) response surface plots showing the impact of time (X_1_), temperature (X_2_), and SLR (X_3_) on the extraction yield of CHP are presented in Figure 2. Pectin yield increased with decreasing extraction time and temperature, since pectin is a polymer of a chain of galacturonic acid units linked with α-(1→4) glycosidic bonds, which might be degraded at higher temperatures and prolonged extraction times [34]. Pectin recovery from pineapple did indeed increase between 70 and 80 °C during extraction, but began to decrease around 90 °C [35]. Given that cocoa bean shells are considerably stronger than pineapple peels, it is unsurprising that CHP requires a higher extraction temperature (105 °C).

High pressures during autoclave extraction can cause deformation and plant membrane breakage, and the pressure transfers to the whole material as a uniform and instant process [36]. Therefore, a shorter duration of extraction is advantageous [36]. Pectin yield did indeed recover after a short period of extraction; however, it started to decline as the extraction time increased, as illustrated in Figure 2D.

Many previous studies used SLR as an independent variable for pectin extraction. The results in Figure 2 show a negative effect between SLR and pectin yield, indicating that an increase in SLR reduced the pectin yield, probably due to difficulties in separation. A lower SLR increased the contact surface area between the plant matrix and the solvent, resulting in a higher pectin yield [31]. Ma et al. [37] investigated the extraction of low-methoxyl pectin from fresh sunflower heads using subcritical water extraction and achieved similar results.

### 3.3. Optimization and Validation of Extraction Conditions

Based on the results, the desired function method was applied to optimize pectin extraction conditions from cocoa husks. The optimal conditions to achieve maximum extraction yield were an extraction time of 5 min, temperature 105 °C, and SLR 20 *w*/*v*. Under these conditions, the predicted maximum yield of CHP was 26.17%, with a desirability value of 1.00. Three independent experiments were performed under optimal conditions to confirm that the predicted values did not deviate significantly from the experimental values. The average experimental value of pectin yield was 26.22 ± 2.03%, and was insignificantly different compared to the predicted value. Therefore, the obtained model was confirmed to be an accurate and reliable tool for predicting the pectin yield from cocoa husks.

The yield (26.22%) obtained in this study was higher than those recorded by previous studies of CHP extraction using nitric acid (11.7%) [38], microwave-assisted (1.9–9.6%) [39], ultrasound-assisted (8.3%) [40], and enzymatic (8.0–13.5%) methods [38]. Therefore, the autoclave extraction of cocoa husks was an appropriate approach to achieve higher pectin yield.

### 3.4. Physicochemical and Structural Characteristics of Pectin

The physicochemical characteristics of CHP obtained under the optimal condition were compared with commercial high-methoxyl pectin (CHMP) and commercial low-methoxyl pectin (CLMP), with the results shown in Table 5.

#### 3.4.1. Product Color, pH, Moisture Content, and Water Activity

Color is an important parameter affecting a product’s final appearance. The lightness (L*), redness (a*), and yellowness (b*) values of the CHP, CHMP, and CLMP samples ranged from 87.52 to 44.81; 1.70 to 111.59; and 9.79 to 23.08, respectively. Significant differences were found among the color values of pectin samples. CHP had a lower L* value but higher a* and b* values than both CHMP and CLMP because the pectin samples were extracted using a different extraction method using a different material. The color values of CHP were predominantly due to high pigment content in the exocarp of cocoa husks which related to tannins and other polyphenolic compounds, which could transfer to the pectin [41]. The pH values of the pectin samples ranged from 3.05 to 5.46, similar to the pH values of pectin from fresh sunflower heads, grapes, and citrus [37,42]. The moisture content and water activity of CHP, CHMP, and CLMP ranged from 4.59 to −10.15% and 0.19 to 0.28, respectively. The moisture contents of the pectin samples were less than 12%, in agreement with the requirement of the Food Chemical Codex [43].

#### 3.4.2. Equivalent Weight, Methoxyl Content (MeO), Anhydrouronic Acid (AUA) Content, Degree of Esterification (DE), Intrinsic Viscosity, and Viscosity–Average Molecular Weight (M_v_)

Pectin AUA and DE are calculated using the equivalent weight, which is a measure of the total free galacturonic acid concentration in pectin. As shown in Table 3, the equivalent weight of CHP (4594.08 mg) was significantly higher than CHMP and CLMP (1388.86 and 1380.33 mg, respectively). The equivalent weight of CHP in this study was also higher than pectin extracted from cocoa peels using a microwave-assisted method (3000 mg). Differences in equivalent weight are affected by extraction conditions such as the pH, acid type, temperature, and the number of free acids available depending on the nature of the pectin source. The gel-forming capabilities of pectin are shown to be higher when the equivalent weight is increased [44].

The methoxyl content (MeO) represents the degree of methylation and can be used to analyze the capacity of pectin to form a gel. Pectin is classified as high-methoxyl pectin (HMP) when it contains >7.12% and as low-methoxyl pectin (LMP) when it has 2.5–7.12% of methoxyl [44]. HMP can form a gel in the presence of sugar and acid, while LMP can form a gel without sugar [45]. The methoxyl contents of CHP and CLMP were 5.08 and 5.08%, respectively, classified as low-methoxyl pectin, while CHMP was classified as high-methoxyl pectin (10.15%).

The amount of anhydrouronic acid (AUA) presents a measure of the purity of the extracted pectin, and it also affects the structure and texture of the pectin gel substance. According to the International Pectin Producers Association (IPPA), the minimal content of AUA in pectin must be 35% [46], while the Food Chemical Codex recommends an AUA content of 65% for pectin used as food additives and for pharmaceutical purposes [43]. In this study, the AUA content of CHP (32.71%) was lower than CHMP (72.69%) and CLMP (41.24%), and below the IPPA and Food Chemical Codex standards, possibly due to the extraction conditions of time, temperature, and pH. Sarah et al. [47] found that AUA pectin content increased with the increasing extraction time.

The degree of esterification (DE) is the number of galacturonic acid groups that are esterified compared to the total number of galacturonic acid groups in pectin. DE is a crucial metric utilized to assess the gelation of pectin. Pectin can be classified into two groups, as LMP with DE less than 50%, and HMP with DE higher than 50% [44]. The DE can be measured using the titration method or using FTIR. Table 3 and Supplementary Figure S1 show that the DE values of CHP, CHMP, and CLMP range between 34.74 and 35.42%; 68.13 and 78.90%; and 26.63 and 46.34%, respectively. The DE values of CHP, CHMP, and CLMP assessed using the two methods showed differences of 0.68, 10.77, and 19.71, respectively. The DE of HCP was higher than the DE values reported for pectin extraction from cocoa pod husk [48]. According to the IPPA, CHP was classified as LMP because it had a DE value lower than 50% [46].

The intrinsic viscosity [ƞ] is a measure of the hydrodynamic volume maintained by macromolecules, which is related to the size and conformation of the macromolecular chains in a solution. A larger intrinsic viscosity indicates a higher molecular weight of the macromolecule, resulting in a better gelling quality [49]. No significant differences were found between the intrinsic viscosity and M_v_ for HCP and CLMP, while significantly lower intrinsic viscosity and M_v_ were observed in CHMP (Table 3). This result indicated that HCP had a competitive advantage in gelling agents and thickeners [50].

#### 3.4.3. Water- and Oil-Holding Capacity (WHC and OHC)

Pectin with higher water-holding capacity (WHC) shows improved textural and sensory properties and reduces the syneresis problems of some food products such as yogurt [51]. WHC refers to the amount of water retained by 1 g of pectin. One gram of CHP, CHMP, and CLMP can hold 11.05, 11.58, and 11.63 g of water, respectively. No significant differences in WHC were found between CHMP and CLMP, while CHMP and CLMP showed higher WHC than CHP. In this study, the WHC of CHP was higher than eggplant-peel pectin (6.02 g/g) and pistachio-green-hull pectin (4.11 g/g) [25]. The oil-holding capacity (OHC) indicates the amount of oil held by 1 g of pectin. OHC is an essential property of hydrocolloids. Pectin with high OHC acts as a good stabilizer and emulsifier in fatty food products [51]. The OHC values of CHP, CHMP, and CLMP were 2.83, 2.48, and 2.28 g/g, respectively, with CHP having a significantly higher OHC than the others. The OHC value of CHP was higher than in eggplant-peel pectin (2.60 g/g) and pistachio-green-hull pectin (2.02 g/g) [25]. According to Elleuch et al. [52], the ionic strength, porosity, structure, and chemical composition of the extracted pectin all affect the WHC and OHC. Our results indicated that CHP could be used in meat products to improve both texture and sensory properties.

#### 3.4.4. Water Solubility Index (WSI)

**Figure 3:**
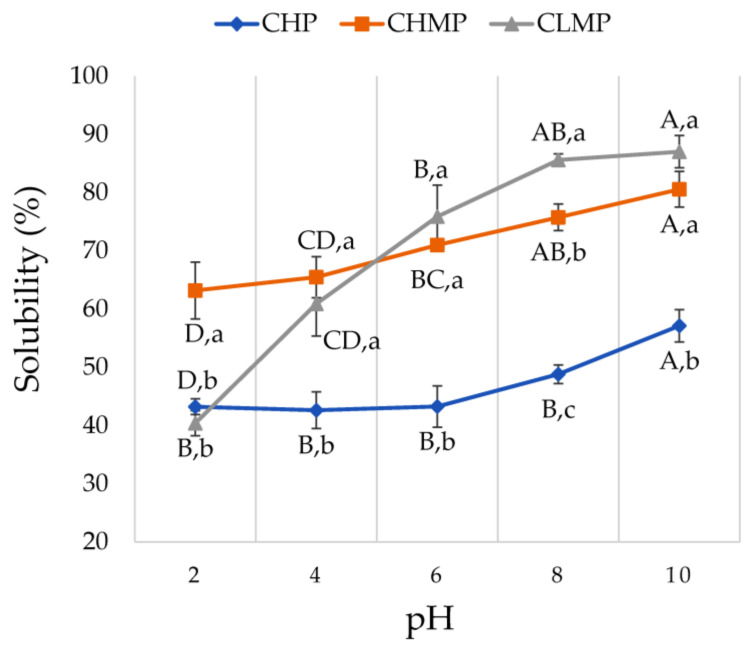
Solubility of cocoa husk pectin (CHP), the commercial high-methoxyl pectin (CHMP) and commercial low-methoxyl pectin (CLMP). Statistical analyses of solubility of pectin samples at different pH are indicated as uppercase letter with significant difference at *p* < 0.05 using one-way analysis of variance (ANOVA) and Duncan’s multiple comparison test. The lowercase letter denotes a significant difference in solubility of different pectin at the same pH using one-way analysis of variance (ANOVA) and Duncan’s multiple comparison test at *p* < 0.05.

The water solubility index (WSI) values of CHP, CHMP, and CLMP are shown in Figure 3. Results indicated that the pectin source and pH significantly affected pectin solubility. The highest solubility was found in CLMP, while CHP showed the lowest solubility. As a result of their increased porosity, smaller particle size, and higher surface area, CLMP and CHMP exhibited a higher degree of solubility compared to CHP. High temperature and pressure also reduced pectin solubility [26]. At pH values of 2 to 10, all pectin samples gave the lowest solubility at pH 2, while solubility increased as pH increased. Similar results were reported by Begum et al. [26], who found that the solubility of jackfruit-waste pectin increased at pH values higher than 4.

#### 3.4.5. Rheological Measurements

**Figure 4:**
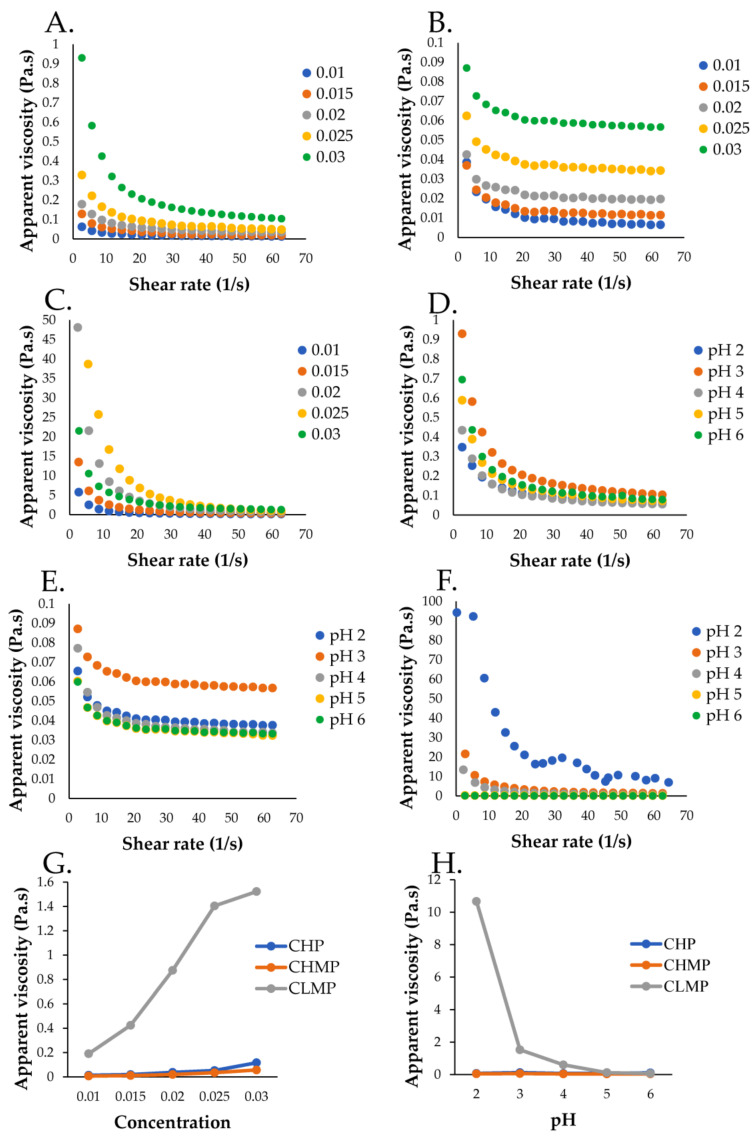
Viscosity–shear rate profiles of (**A**) CHP, (**B**) CHMP, and (**C**) CLMP solutions at concentration 1.0–3.0% (*w*/*v*). (**G**) Effect of concentration on the viscosities of CHP, CHMP, and CLMP solution (shear rate = 50 s^−1^). Viscosity–shear rate profiles of 3% (*w*/*v*). (**D**) CHP, (**E**) CHMP, and (**F**) CLMP solutions at pH 2–6. (**H**) Effect of pH on the viscosities of 3% (*w*/*v*) CHP, CHMP, and CLMP solutions (shear rate = 50 s^−1^): cocoa husk pectin (CHP), the commercial high-methoxyl pectin (CHMP), and commercial low-methoxyl pectin (CLMP).

The flow behaviors of CHP, CHMP, and CLMP at different concentrations (1.0–3.0% *w*/*v*) between 1 and 100 s^−1^ shear rates at 25 °C are presented in Figure 4A–C. All pectin solutions exhibited pseudoplastic or shear-thinning behavior, in which the viscosity decreased with the increasing shear rate [53]. The pectin solutions followed Newtonian fluid behavior at high shear rates. The pseudoplastic region of the pectin solution was stronger at higher concentrations than at lower concentrations. This was because intermolecular networks formed between the pectin chains. Similar flow behaviors were also observed in durian pectin solution [10] and sunflower head pectin solution [53]. Figure 4G shows that the viscosity of pectin solutions increased with increasing pectin concentration, while the viscosity of CLMP solutions was higher than CHP and CHMP solutions due to the higher number of straighter pectin chains in CLMP, which enhanced aggregation [53]. All the pectin solutions exhibited shear thinning behavior at pH 2–6, while viscosity decreased with increasing shear rate (Figure 4D–F). The shear thinning properties of the pectin solutions were more apparent at lower pH than at higher pH values, with the effect of pH on pectin solution viscosity at 50 s^−1^ being shown in Figure 4H. The viscosity of pectin solutions is significantly influenced by the pH value, which modifies the charge distribution along the pectin chains [54]. The findings indicated that when the pH increased, the viscosities of pectin solutions declined. Other studies also found that CLMP solutions had higher viscosities than CHP and CHMP solutions [10,53].

**Figure 5:**
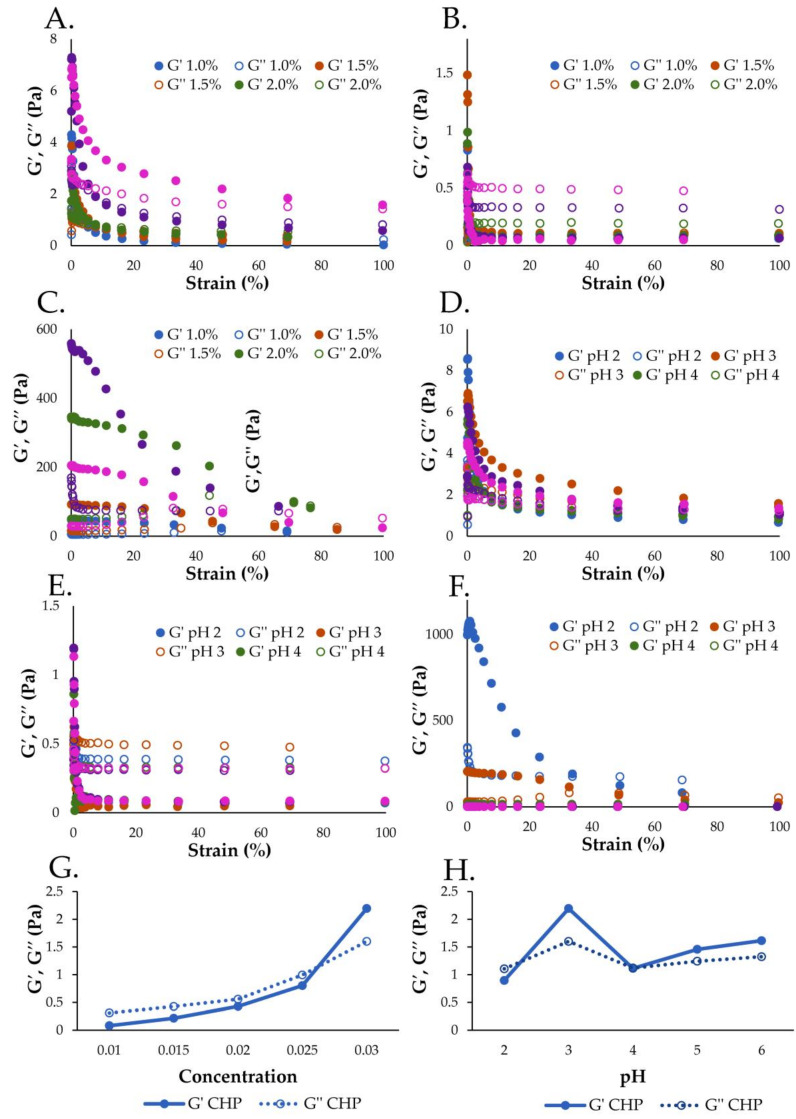
Amplitude sweep studies of (**A**) CHP, (**B**) CHMP, and (**C**) CLMP solutions at concentrations 1.0–3.0% (*w*/*v*). (**G**) Effect of concentration on the viscoelastic properties of CHP solutions (strain = 50%). Viscosity-shear rate profiles of 3% (*w*/*v*). (**D**) CHP, (**E**) CHMP, and (**F**) CLMP solutions at pH 2.0–6.0. (**H**) Effect of pH on the viscoelastic properties of 3% (*w*/*v*) CHP solutions (strain = 50%): cocoa husk pectin (CHP), the commercial high-methoxyl pectin (CHMP) and commercial low-methoxyl pectin (CLMP).

Figure 5A–C present the viscoelastic properties of CHP, CHMP, and CLMP solutions at different concentrations (1.0–3.0% *w*/*v*). The results demonstrated that the viscoelastic characteristics of pectin solutions improved at increasing concentrations, which explained the enhanced creation of cross-linking junction zones between pectin chains [10]. The viscoelastic properties of CHP solutions as a function of pectin concentration are shown in Figure 5G. The G′ value increased at increasing concentrations. At concentrations of 1.0, 1.5, 2.0, and 2.5% *w*/*v*, a liquid-like gel was formed (G′ < G″), while a soft gel was generated at 3.0% *w*/*v* (G′ > G″). The pH value is a critical factor that affects the gelling mechanisms of pectin. Therefore, the effect of pH on the viscoelastic properties of CHP solutions was assessed, with results shown in Figure 5D–F. At pH 3, CHP showed the best gel-like behavior (G′ > G″), while CHP solution at pH 4–6 showed predominantly gel-like behavior (G′ > G″), and pectin solutions at pH 5 and 6 had better viscoelastic properties than at pH 4. Figure 5H shows that G′ and G″ values increased from pH 2, and were highest at pH 3. The G′ and G″ values decreased when pH values increased from 3 to 4 and then slightly increased at pH 4 to 6. The highest viscoelastic properties at pH 3 were explained by the stabilization of pectin chain intermolecular interactions by carboxyl groups protonated at pH 3 [54], with a decrease in viscoelastic properties at pH 4 due to the increased degree of dissociation of carboxyl groups in the pectin molecules [10]. Based on the results, CHP could be used as a gelling agent and thickener in food products due to its pseudoplastic and viscoelastic properties.

### 3.5. Pesticides and Heavy Metal Analysis of Raw Materials and CHP

Pesticides are used in cocoa plantations to enhance crop production [55,56], with residues reported in soil, cocoa beans, cocoa leaves, and cocoa shells [57,58,59,60]. In this study, residues of organophosphate, organochlorine, pyrethroid, and carbamate were below the detection limit of 0.005 mg/kg in cocoa husk samples (Supplementary Materials Table S1) compared to other cultivation areas. The pesticide compounds determined in this study are shown in the Supplementary Table S1. This discrepancy was due to the area of plantation, practices, and regulations as factors associated with the presence of pesticides in food crops [61]. Concentrations of the heavy metals cadmium (Cd), arsenic (As), and mercury (Hg) in cocoa husks were 0.521 ± 0.059 mg/kg, 0.042 ± 0.005 mg/kg, and 0.072 ± 0.044 mg/kg, respectively, while lead (Pb) levels were below the limit of quantification (<0.005 mg/kg). The accumulation of heavy metals in plants depends on the cultivar, growth stage, and heavy metal content in the soil. The cocoa plant is a heavy metal accumulator, especially for cadmium. Variations in heavy metal contents accumulated in different parts of cocoa plants were reported [62]. After extraction, the pectin contained 0.570 ± 0.007 mg/kg of cadmium at a similar level to the cocoa husk, while the level of arsenic reduced to 0.028 ± 0.001 mg/kg, and the levels of lead and mercury reduced to below the detection limit (Supplementary Materials Tables S2 and S3). The reduction of heavy metals in the extracted pectin was due to losses during the washing process. The level of heavy metals in CHP complied with the current maximum limit for pectin (E 440i) according to Regulation (EU) No 231/2012 [63], at 3 mg/kg for arsenic, 1 mg/kg for lead, and 1 mg/kg for mercury and cadmium. Thus, CHP can be considered for potential application as a food additive.

### 3.6. Genotoxicity Determination Using the Bacterial Reverse Mutation Test (Ames Test)

Pectin derived from citrus fruits and apples is safe for consumption and is distributed throughout food sectors. However, there are no reports on the genotoxicity of pectin derived from cocoa husks. The genotoxicity of CHP was tested using the bacterial reverse mutation test (Ames test) following guidelines from the Organization for Economic Co-operation and Development (OECD) [27] to promote usage as a food ingredient. Five strains of *Salmonella Typhimurium* were used to detect various types of DNA damage. The S9 extract was also employed to distinguish between direct and indirect mutagens in the samples. The Ames assay compared the more revertant colonies with the negative control to indicate DNA mutation. Results in Table 6 show that the number of revertant colonies in bacteria exposed to CHP without the S9 extract from 10 to 2000 µg/plate did not increase compared to the negative control. The mutagenic ratio (MR) was negative, suggesting that CHP was not a direct mutagen, while the positive control (mutagens) showed high numbers of revertant colonies, and the MR was positive. Table 7 shows the number of revertant colonies for bacteria exposed to CHP with the S9 extract. Results were consistent with Table 6, showing that the number of revertant colonies did not increase compared to the negative control, even at the highest dose (2000 µg/plate). The MR was negative, implying that CHP was not an indirect mutagen. Our data were consistent with previous reports showing that pectin derived from plant sources, including pumpkin and carrot, were also devoid of genotoxicity [64,65].

## 4. Conclusions

An autoclave approach was utilized to optimize the conditions for extracting pectin from cocoa husks, a significant food waste product. The optimal conditions were 105 °C for 5 min with SLR at 20 *w*/*v*. Under these extraction conditions, high levels of CHP were recovered at 26.22%. The obtained CHP was characterized as low-methoxyl pectin (degree of esterification 34.74% and methoxyl content 5.08%). The color, pH, moisture content, and water activity of CHP differed from CHMP and CLMP but were within the requirements of the Food Chemical Codex. Results demonstrated that this autoclave approach to extract pectin from cocoa husks would not only help to manage the disposal of cocoa husk waste, but CHP also showed promise as an alternative source of low-methoxyl pectin and could be used as a gelling agent or thickener in food. Finally, further research could be performed for improving the pectin purity and its potential use in pharmacological and medical applications.

## Figures and Tables

**Table 1:** Box–Behnken design matrix with independent variables and their actual levels.

Factors	Unit	Actual Levels		
		−1	0	1
(X_1_) Extraction time	min	5	20	40
(X_2_) Extraction temperature	°C	105	120	135
(X_3_) Solid–liquid ratio (SLR)	*w*/*v*	10	20	30

**Table 2:** Uncoded Box–Behnken design (BBD) of independent variables obtained from Table 1.

Run	X_1_: Extraction Time (min)	X_2_: Temperature (°C)	X_3_: Solid–Liquid Ratio (SLR) (*w*/*v*)
1	40	120	10
2	20	105	10
3	5	120	10
4	40	105	20
5	20	120	20
6	20	105	30
7	20	120	20
8	5	120	30
9	20	135	10
10	5	135	20
11	40	135	20
12	20	135	30
13	40	120	30
14	20	120	20
15	5	105	20

**Table 3:** Box–Behnken design of independent variables derived from Table 1, experimental and predicted yield of CHP.

Run	X_1_: Time (min)	X_2_: Temperature (°C)	X_3_: SLR (*w*/*v*)	Experimental Yield (%)	Predicted Yield (%)
1	40	120	10	14.9	14.91
2	20	105	10	22.1	23.84
3	5	120	10	20.9	18.90
4	40	105	20	22.05	20.56
5	20	120	20	9.55	9.92
6	20	105	30	19.25	19.00
7	20	120	20	10.6	9.92
8	5	120	30	15.25	15.53
9	20	135	10	5.85	6.11
10	5	135	20	6.8	8.51
11	40	135	20	1.85	1.63
12	20	135	30	2.05	0.32
13	40	120	30	5.3	7.02
14	20	120	20	9.6	9.92
15	5	105	20	26.15	26.17

**Table 4:** The results of analysis of variance (ANOVA) for regression model of CHP yield.

Source	Sum of Squares	DF	Mean Square	F-Value	*p*-Value	Significance
Model	850.49	9	94.50	24.78	0.0013	**
X_1_	78.13	1	78.13	20.49	0.0062	**
X_2_	662.75	1	662.75	173.81	<0.0001	***
X_3_	62.90	1	62.90	16.50	0.0097	**
X_1_X_2_	0.4091	1	0.4091	0.1073	0.7565	
X_1_X_3_	5.16	1	5.16	1.35	0.2974	
X_2_X_3_	0.2256	1	0.2256	0.0592	0.8175	
X_1_^2^	44.35	1	44.35	11.63	0.0190	
X_2_^2^	5.87	1	5.87	1.54	0.2699	
X_3_^2^	4.76	1	4.76	1.25	0.3147	
Residual Error	19.07	5	3.81			
Lack of fit	18.36	3	6.12	17.45	0.0547	ns
Pure Error	0.7017	2	0.3508			
Total	869.56	14				
R^2^	0.9781					
Adj R^2^	0.9386					
Pred R^2^	0.6559					

Experimental data shown as analysis of variance (ANOVA) for response surface quadratic model. X_1_: extraction time; X_2_: extraction temperature; X_3_: solid–liquid ratio (SLR). ** and *** show a significant difference determined using Design-Expert. ** Significant at *p* < 0.01; *** Significant at *p* < 0.001; and ns: not significant.

**Table 5:** Physicochemical properties of cocoa husk pectin (CHP), commercial high-methoxyl pectin (CHMP), and commercial low-methoxyl pectin (CLMP).

Parameters	Pectin Sample		
	CHP	CHMP	CLMP
Color			
L*	44.81 ± 0.13 ^c^	75.39 ± 0.13 ^b^	87.52 ± 0.00 ^a^
a*	11.59 ± 0.02 ^a^	6.19 ± 0.05 ^b^	1.70 ± 0.00 ^c^
b*	23.08 ± 0.02 ^a^	16.38 ± 0.07 ^b^	9.79 ± 0.16 ^c^
pH	5.46 ± 0.02 ^a^	3.05 ± 0.07 ^c^	4.51 ± 0.04 ^b^
Moisture content (%)	8.27 ± 0.37 ^b^	10.15 ± 0.08 ^a^	4.59 ± 0.08 ^c^
Water activity (a_w_)	0.21 ± 0.00 ^b^	0.19 ± 0.00 ^c^	0.28 ± 0.00 ^a^
Equivalent weight	4594.08 ± 414.92 ^a^	1388.86 ± 4.20 ^b^	1380.33 ± 8.06 ^b^
MeO (%)	5.08 ± 0.12 ^b^	10.56 ± 0.06 ^a^	5.01 ± 0.36 ^b^
AUA (%)	32.71 ± 1.06 ^c^	72.69 ± 1.58 ^a^	41.24 ± 2.08 ^b^
DE (%) using titration	35.42 ± 2.08 ^b^	68.13 ± 1.54 ^a^	26.63 ± 1.47 ^c^
DE (%) using FT-IR	34.74 ± 0.06 ^c^	78.90 ± 1.60 ^a^	46.34 ± 3.46 ^b^
[ƞ]	3.60 ± 0.30 ^a^	2.38 ± 0.11 ^b^	3.29 ± 0.01 ^a^
M_v_ (kDa)	79.38 ± 8.99 ^a^	45.00 ± 2.79 ^b^	69.97 ± 0.27 ^a^
WHC (g/g)	11.05 ± 0.07 ^b^	11.58 ± 0.05 ^a^	11.63 ± 0.02 ^a^
OHC (g/g)	2.83 ± 0.14 ^a^	2.48 ± 0.02 ^b^	2.28 ± 0.03 ^b^

All data are demonstrated as mean ± standard deviation (SD) of three independent sets of samples analyzed in triplicate (*n* = 3). Statistical analyses of physicochemical properties are indicated as lowercase letter with significant difference at *p* < 0.05 using one-way analysis of variance (ANOVA) and Duncan’s multiple comparison test. MeO: methoxyl content; AUA: anhydrouronic content; DE: degree of esterification; [ƞ]: intrinsic viscosity; M_v_: viscosity-average molecular weight; WHC: water-holding capacity; OHC: oil-holding capacity.

**Table 6:** Mutagenicity effects of CHP on five *S. typhimurium* strains without S9 extract (-S9).

Doses (µg/plate)	TA98		TA100		TA102		TA1535		TA1537	
	Revertant Colonies	MR	Revertant Colonies	MR	Revertant Colonies	MR	Revertant Colonies	MR	Revertant Colonies	MR
Neg	71.26 ± 0.18	1.00 (−)	72.45 ± 2.11	1.00 (−)	203.67 ± 5.44	1.00 (−)	20.00 ± 1.63	1.00 (−)	39.67 ± 2.05	1.00 (−)
10	74.00 ± 4.55	1.1 (−)	75.67 ± 6.94	1.2 (−)	225.33 ± 10.34	1.11 (−)	16.00 ± 1.41	0.80 (−)	39.33 ± 1.70	0.99 (−)
100	85.67 ± 1.70	1.2 (−)	72.00 ± 2.45	1.1 (−)	228.00 ± 12.75	1.12 (−)	18.00 ± 1.41	0.90 (−)	38.00 ± 2.16	0.96 (−)
500	71.67 ± 2.36	1.0 (−)	76.67 ± 3.30	1.2 (−)	206.00 ± 6.68	1.01 (−)	20.00 ± 0.82	1.00 (−)	38.00 ± 2.83	0.96 (−)
1000	93.33 ± 2.87	1.3 (−)	70.67 ± 3.09	1.1 (−)	219.00 ± 15.75	1.08 (−)	19.33 ± 0.47	0.97 (−)	35.67 ± 6.65	0.90 (−)
2000	83.00 ± 4.24	1.2 (−)	78.33 ± 5.44	1.2 (−)	209.67 ± 9.98	1.03 (−)	18.67 ± 1.70	0.93 (−)	40.00 ± 1.63	1.01 (−)
4-NQO	715.33 ± 42.91	10.2 (+)								
NaN_3_			1013.33 ± 10.50	15.8 (+)			254.33 ± 20.76	12.72 (+)		
MMC					978.33 ± 30.00	4.80 (+)				
9-AA									852.00 ± 41.04	21.48 (+)

Results are shown as mean ± standard deviation (SD) of three experiments (*n* = 3). Neg (negative control) is dimethyl sulfoxide, which *is* used as a solvent control. (−) indicates the mutagenicity ratio (MR) is ≤1, and (+) indicates the mutagenicity ratio (MR) is ≥2.

**Table 7:** Mutagenicity effects of CHP on five *S. typhimurium* strains with S9 extract (+S9).

Doses (µg/plate)	TA98		TA100		TA102		TA1535		TA1537	
	Revertant Colonies	MR	Revertant Colonies	MR	Revertant Colonies	MR	Revertant Colonies	MR	Revertant Colonies	MR
Neg	82.33 ± 4.50	1.00 (−)	71.33 ± 24.64	1.00 (−)	298.00 ± 3.56	1.00 (−)	18.33 ± 1.25	1.00 (−)	33.00 ± 2.94	1.00 (−)
10	88.67 ± 1.25	1.1 (−)	85.00 ± 7.35	1.2 (−)	321.00 ± 16.99	1.08 (−)	18.00 ± 1.41	0.98 (−)	31.00 ± 0.82	0.94 (−)
100	116.33 ± 9.10	1.4 (−)	77.67 ± 5.25	1.1 (−)	271.33 ± 19.15	0.91 (−)	21.33 ± 1.25	1.16 (−)	29.00 ± 1.41	0.88 (−)
500	116.00 ± 11.52	1.4 (−)	75.67 ± 4.11	1.1 (−)	259.67 ± 11.26	0.87 (−)	23.00 ± 3.27	1.25 (−)	28.33 ± 2.62	0.86 (−)
1000	129.33 ± 11.44	1.6 (−)	65.67± 3.68	0.9 (−)	304.00 ± 5.72	1.02 (−)	21.00 ± 0.82	1.15 (−)	36.33 ± 1.89	1.10 (−)
2000	130.00 ± 6.98	1.6 (−)	71.33 ± 1.25	1.0 (−)	301.33 ± 27.15	1.01 (−)	19.00 ± 1.41	1.04 (−)	30.33 ± 3.40	0.92 (−)
2-AA	1096.33 ± 72.97	13.3 (+)	1190.33 ± 33.33	16.7 (+)	1024.00 ± 11.52	3.44 (+)	303.67 ± 12.92	16.56 (+)	237.33 ± 6.13	7.19 (+)

Results are shown as mean ± standard deviation (SD) of three experiments (*n* = 3). Neg (negative control) is dimethyl sulfoxide, which used as a solvent control. (−) indicates the mutagenicity ratio (MR) is ≤1, and (+) indicates the mutagenicity ratio (MR) is ≥2.

## Data Availability

The original contributions presented in the study are included in the article/Supplementary Materials, further inquiries can be directed to the corresponding author.

## Supplementary Materials

The following supporting information can be downloaded at https://www.mdpi.com/article/10.3390/foods13050669/s1, Supplementary Table S1: Pesticide content of the cocoa husks used in the present study.; Supplementary Table S2: Heavy metals content of the cocoa husks used in the present study, and Supplementary Table S3: Heavy metals content of cocoa husk pectin (CHP) obtained from the present study. Supplementary Figure S1: The Fourier Transform Infrared Spectra (FTIR) of (A) cocoa husk pectin (CHP), (B) commercial high-methoxyl pectin (CHMP) and (C) commercial low-methoxyl pectin (CLMP).
