# Supplementary material for: High Pectin Recovery from Cocoa Husks Using an Autoclave Approach: An Analysis of Its Physicochemical, Structural, and Genotoxicity Properties
Source: Foods. 2024 Feb 22;13(5):669. doi: 10.3390/foods13050669 (PMC10931213; doi:10.3390/foods13050669)

Supplementary materials

High Pectin Recovery from Cocoa Husks Using an Autoclave Approach: An Analysis of Its Physicochemical, Structural, and Genotoxicity Properties

Thanaporn Pinkaew [1], Woorawee Inthachat [2], Chanakan Khemthong [2], Varongsiri Kemsawasd [2], Nattira On-Nom [2,*,†] and Piya Temviriyanukul [2,†]

[1] Master of Science Program in Toxicology and Nutrition for Food Safety, Institute of Nutrition, Mahidol University, Nakhon Pathom 73170, Thailand; thanaporn.pik@student.mahidol.ac.th

[2] Food and Nutrition Academic and Research Cluster, Institute of Nutrition, Mahidol University, Salaya, Phuttamonthon, Nakhon Pathom 73170, Thailand; woorawee.int@mahidol.ac.th (W.I.); chanakan.khe@mahidol.ac.th (C.K.); varongsiri.kem@mahidol.ac.th (V.K.); piya.tem@mahidol.ac.th (P.T.)

* Correspondence: nattira.onn@mahidol.ac.th

† These authors contributed equally to this work.

Supplementary Table S1:

Pesticide content of the cocoa husks used in the present study.

Pesticide	Unit	Test result	LOD	LOQ	Method
Organochlorine					In-house method TPTFS-229TM based on AOAC (2019), 2007.01
Aldrin	mg/kg	Not found	0.005	0.01	
alpha-BHC	mg/kg	Not found	0.005	0.01	
beta-BHC	mg/kg	Not found	0.005	0.01	
delta-BHC	mg/kg	Not found	0.005	0.01	
gamma-BHC	mg/kg	Not found	0.005	0.01	
cis-Chlordane	mg/kg	Not found	0.005	0.01	
trans-Chlordane	mg/kg	Not found	0.005	0.01	
o,p′-DDT	mg/kg	Not found	0.005	0.01	
p,p′-DDT	mg/kg	Not found	0.005	0.01	
Dicofol	mg/kg	Not found	0.005	0.01	
Dieldrin	mg/kg	Not found	0.005	0.01	
Endosulfan	mg/kg	Not found	0.005	0.01	
Endosulfan I	mg/kg	Not found	0.005	0.01	
Endosulfan II	mg/kg	Not found	0.005	0.01	
Endosulfan sulfate	mg/kg	Not found	0.005	0.01	
Endrin	mg/kg	Not found	0.005	0.01	
Heptachlor	mg/kg	Not found	0.005	0.01	
Heptachlor-epoxide	mg/kg	Not found	0.005	0.01	
Methoxychlor	mg/kg	Not found	0.005	0.01	
o,p′-DDE	mg/kg	Not found	0.005	0.01	
p,p′-DDE	mg/kg	Not found	0.005	0.01	
o,p′-DDD	mg/kg	Not found	0.005	0.01	
p,p′-DDD	mg/kg	Not found	0.005	0.01	
Mirex	mg/kg	Not found	0.005	0.01	
Endrin ketone	mg/kg	Not found	0.005	0.01	
Hexachlorobenzene	mg/kg	Not found	0.005	0.01	

Supplementary Table S1 (Cont.):

Pesticide content of the cocoa husks used in the present study.

Pesticide	Unit	Test result	LOD	LOQ	Method
**Organophosphate**					In-house method TPTFS-229TM based on AOAC (2019), 2007.01
Acephate	mg/kg	Not found	0.005	0.01	
Azinphos-ethyl	mg/kg	Not found	0.005	0.01	
Azinphos-methyl	mg/kg	Not found	0.005	0.01	
Chlorpyrifos	mg/kg	Not found	0.005	0.01	
Chlorpyrifos-methyl	mg/kg	Not found	0.005	0.01	
Dichlorvos	mg/kg	Not found	0.005	0.01	
Diazinon	mg/kg	Not found	0.005	0.01	
Disulfoton	mg/kg	Not found	0.005	0.01	
Dicrotophos	mg/kg	Not found	0.005	0.01	
Dimethoate	mg/kg	Not found	0.005	0.01	
EPN	mg/kg	Not found	0.005	0.01	
Ethion	mg/kg	Not found	0.005	0.01	
Fenitrothion	mg/kg	Not found	0.005	0.01	
Malathion	mg/kg	Not found	0.005	0.01	
Methamidophos	mg/kg	Not found	0.005	0.01	
Methidathion	mg/kg	Not found	0.005	0.01	
Mevinphos	mg/kg	Not found	0.005	0.01	
Monocrotophos	mg/kg	Not found	0.005	0.01	
Omethoate	mg/kg	Not found	0.005	0.01	
Parathion-ethyl	mg/kg	Not found	0.005	0.01	
Parathion-methyl	mg/kg	Not found	0.005	0.01	
Phosalone	mg/kg	Not found	0.005	0.01	
Pirimiphos-ethyl	mg/kg	Not found	0.005	0.01	
Pirimiphos-methyl	mg/kg	Not found	0.005	0.01	
Profenofos	mg/kg	Not found	0.005	0.01	
Prothiophos	mg/kg	Not found	0.005	0.01	
Triazophos	mg/kg	Not found	0.005	0.01	
Phosphamidon	mg/kg	Not found	0.005	0.01	

Supplementary Table S1 (Cont.):

Pesticide content of the cocoa husks used in the present study.

Pesticide	Unit	Test result	LOD	LOQ	Method
**Pyrethroid**					In-house method TPT-FS-229TM based on AOAC (2019), 2007.01
Bifenthrin	mg/kg	Not found	0.005	0.01	
Cyfluthrin	mg/kg	Not found	0.005	0.01	
Cypermethrin	mg/kg	Not found	0.005	0.01	
Deltamethrin	mg/kg	Not found	0.005	0.01	
Fenpropathrin	mg/kg	Not found	0.005	0.01	
Fenvalerate	mg/kg	Not found	0.005	0.01	
lambda-Cyhalothrin	mg/kg	Not found	0.005	0.01	
Permethrin	mg/kg	Not found	0.005	0.01	
**Carbamate**					In-house method TPT-FS-241TM based on AOAC (2019), 2007.01
Aldicarb	mg/kg	Not found	0.006	0.01	
Aldicarb sulfone	mg/kg	Not found	0.006	0.01	
Aldicarb sulfoxide	mg/kg	Not found	0.006	0.01	
Carbofuran	mg/kg	Not found	0.006	0.01	
Carbofuran-3-hydroxy	mg/kg	Not found	0.006	0.01	
Carbaryl	mg/kg	Not found	0.006	0.01	
Methiocarb	mg/kg	Not found	0.006	0.01	
Methomyl	mg/kg	Not found	0.006	0.01	
Oxamyl	mg/kg	Not found	0.006	0.01	
Propoxur	mg/kg	Not found	0.006	0.01	
Fenobucarb	mg/kg	Not found	0.006	0.01	
Promecarb	mg/kg	Not found	0.006	0.01	
Isoprocarb	mg/kg	Not found	0.006	0.01	
Meltolcarb	mg/kg	Not found	0.006	0.01	

Supplementary Table S2:

Heavy metals content of the cocoa husks used in the present study.

Heavy metal	Unit	Test result	LOD	LOQ	Method
Arsenic (As)	mg/kg	0.042 ± 0.005	0.002	0.005	In-house method TPT-FS-282TM based on AOAC (2019), 2015.01
Lead (Pb)	mg/kg	<0.005	0.002	0.005	
Cadmium (Cd)	mg/kg	0.521 ± 0.059	0.002	0.005	
Mercury (Hg)	mg/kg	0.072 ± 0.044	0.002	0.005	

Supplementary Table S3:

Heavy metals content of cocoa husk pectin (CHP) obtained from the present study.

Heavy metal	Unit	Test result	LOD	LOQ	Method
Arsenic (As)	mg/kg	0.028 ± 0.001	0.002	0.005	In-house method TPT-FS-282TM based on AOAC (2019), 2015.01
Lead (Pb)	mg/kg	Not found	0.002	0.005	
Cadmium (Cd)	mg/kg	0.570 ± 0.007	0.002	0.005	
Mercury (Hg)	mg/kg	Not found	0.002	0.005	

Supplementary Figure S1:

The Fourier Transform Infrared Spectra (FTIR) of (A) cocoa husk pectin (CHP), (B) commercial high-methoxyl pectin (CHMP) and (C) commercial low-methoxyl pectin (CLMP).

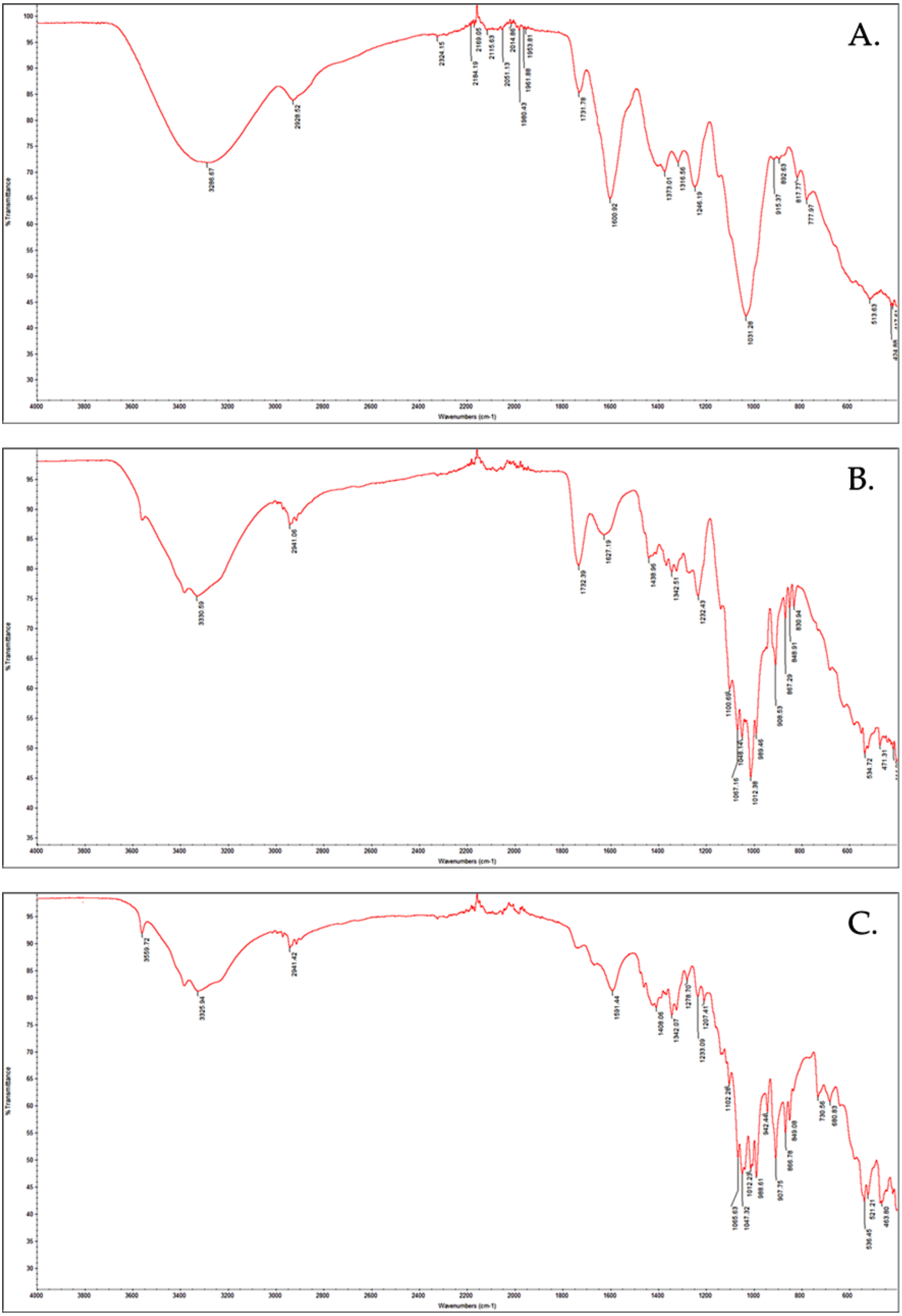

Supplement: Supplementary file 1 [file foods-13-00669-s001.zip › foods-2875540-supplementary.pdf]
